# Intercomparison of radiosensitization induced by gold and iron oxide nanoparticles in human glioblastoma cells irradiated by 6 MV photons

**DOI:** 10.1038/s41598-022-13368-x

**Published:** 2022-06-10

**Authors:** Danieli B. Guerra, Elisa M. N. Oliveira, Amanda R. Sonntag, Patricia Sbaraine, Andre P. Fay, Fernanda B. Morrone, Ricardo M. Papaléo

**Affiliations:** 1 Interdisciplinary Center of Nanoscience and Micro-Nanotechnology, School of Technology, Pontifical Catholic University of Rio Grande do Sul, Porto Alegre, 90619-900 Brazil; 2 Division of Radiotherapy, São Lucas Hospital of PUCRS, Porto Alegre, 90610-000 Brazil; 3 School of Medicine, Pontifical Catholic University of Rio Grande do Sul, Porto Alegre, 90619-900 Brazil; 4 School of Health and Life Sciences, Pontifical Catholic University of Rio Grande do Sul, Porto Alegre, 90619-900 Brazil

**Keywords:** Nanoscience and technology, Cancer therapy

## Abstract

In this work, an intercomparison of sensitization effects produced by gold (GNP) and dextran-coated iron oxide (SPION-DX) nanoparticles in M059J and U87 human glioblastoma cells was performed using 6 MV-photons. Three variables were mapped: the nanoparticle material, treatment concentration, and cell radiosensitivity. For U87, GNP treatments resulted in high sensitization enhancement ratios (SER$_{10\%}$ up to 2.04). More modest effects were induced by SPION-DX, but still significant reductions in survival were achieved (maximum SER$_{10\%}=1.61$ ). For the radiosensitive M059J, sensitization by both NPs was poor. SER$_{10\%}$ increased with the degree of elemental uptake in the cells, but not necessarily with treatment concentration. For GNP, where exposure concentration and elemental uptake were found to be proportional, SER$_{10\%}$ increased linearly with concentration in both cell lines. For SPION-DX, saturation of sensitization enhancement and metal uptake occurred at high exposures. Fold change in the $\alpha /\beta$ ratios extracted from survival curves are reduced by the presence of SPION-DX but strongly increased by GNPs , suggesting that sensitization by GNPs occurs mainly via promotion of lethal damage, while for SPION-DX repairable damage dominates. The NPs were more effective in eliminating the radioresistant glioblastoma cells, an interesting finding, as resistant cells are key targets to improve treatment outcome.

## Introduction

Glioblastoma multiform (GBM) is one of the most lethal malignancies with a 5-year survival rate of 5.5% and a median survival rate of less than 15 months^1^. It is categorized as a grade IV astrocytic glioma, and the most common brain malignancy, accounting for more than 60% of all brain tumours in adults^1,2^. GBM is particularly difficult to treat because of the intrusive penetration of isolated cells into adjoining tissues, preventing the complete surgical removal from the brain^3^. The invasive, infiltrative disease component is the ultimate cause of recurrence, resistance, and death^4^. Traditional GBM therapies include surgery, chemotherapy, and radiotherapy (RT)^2,3,5^. The current standard RT for GBM management consists of 60 Gy of high energy X-rays in 2 Gy/day fractions^6^. As the dose required to eradicate GBM is high, it can trigger side effects in normal brain tissues around the tumor site, including the risk of necrosis^3,5,6^. Therefore, in spite of huge advances in beam delivery methods, the challenge to increase the therapeutic effectiveness of RT, while sparing surrounding healthy tissues remains. One strategy involves the use of radiosensitizers.

In the last years, there has been an increasing interest in utilizing nanoparticles (NPs) with high atomic number (Z), such as metal-based NPs , as radiosensitizers in cancer treatment^7–9^. Originally, the rationale for using high-*Z* materials was based on their energy absorption properties^10^. The cross-section of the photoelectric effect which governs the probability of photon absorption is proportional to $\approx Z^4$. Thus in principle a larger local dose could be deposit in biological tissue near the location of high-*Z* nanoparticles. Indeed, Monte Carlo simulations indicate pronounced enhancement effects for NPs of small radii and made of heavy elements^11–13^. However, as interaction cross sections decrease with photon energy, this effect would only be significant for low energy (keV) X-rays and negligible dose enhancements would be expected at clinical photon energies reaching several MeV^11–13^. In spite of that, numerous in vitro studies have reported significant radiosensitization effects of nanoparticles, specially those made of Au, when MV X-rays  are used^14–18^. Reported radiosensitization enhancement effects are usually larger than the predictions for the corresponding maximal physical dose increase. Therefore, other mechanisms must be involved in the process, including chemical and biological effects triggered by the presence of the NPs, which are yet not properly understood^7,9,19^. Moreover, a large variability in sensitization ratios is found among different studies, even when conducted with apparently similar conditions^9^. Results are far from being conclusive, and substantial controversy remains. There are a number of variables that need to be controlled and their role fully understood (including type of cell line, NP material and coating, treatment concentration and incubation times, NPs spatial distribution within cells, irradiation parameters, and the various biological protocols)^8,20–23^ in order to allow successful translation of NP-enhanced radiotherapy into the clinics.

Here we investigate sensitization effects of gold nanoparticles (by far the most investigated nanoparticulate system in cancer nanotechnology due to their high Z, unique physical properties and excellent biocompatibility^24^) and superparamagnetic iron oxide nanoparticles (SPION), which in spite of their relatively low Z are excellent candidates for theragnostic agents in MRI-guided radiotherapy^25–28^. We provide an intercomparison of radiosensitization effects produced by the two NPs at different treatment concentrations in radiosensitive (M059J) and radioresistent (U87) human glioblastoma cell lines, showing that the combination of NPs with radiotherapy is more effective in eliminating the radioresistant strain. We observed high enhancement factors and significant changes in the shape of the survival curve for Au treatments and more modest effects for SPIONs, and demonstrate that the sensitization enhancement ratio increases linearly with the actual degree of metal internalization in the cells, but not necessarily with the concentration of NPs during incubation.

## Results and discussion

### Nanoparticle characterization

**Figure 1:**
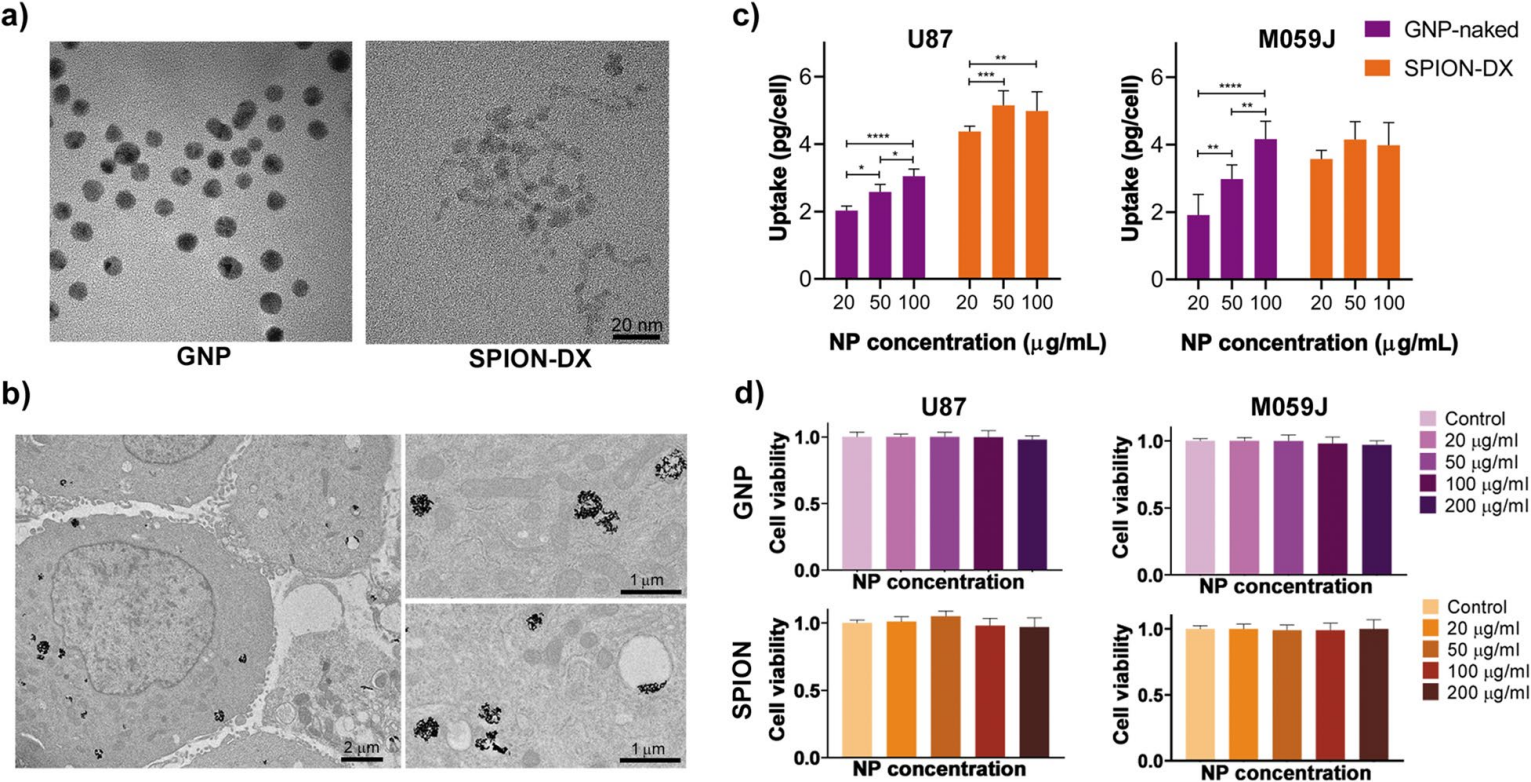
Nanoparticle characteristics and interaction with cells. (**a**) TEM images of GNPs  (left) and SPION-DX (right). The scale bar applies to both images. (**b**) TEM micrographs of U87 cells exposed to $50\,\upmu$/mL GNPs showing NPs (black spots) localized within the cytoplasm. On the right, magnified TEM images from the same cell, showing the NP aggregates within vesicles. (**c**) Average metal (Au or Fe) uptake (in units of $10^{-12}$g per cell) determined using ICP-MS for U87 (left) and M059J (right) cells incubated for 24 h with different concentrations of GNPs  and SPION-DX. *p*-values are presented as: *$p < 0.05$; **$p < 0.01$; ***$p < 0.001$; ****$p < 0.0001$. (**d**) MTT cell viability assay for U87 (left) and M059J (right) GBM cells after 24 h incubation with GNPs and SPION-DX at treatment concentrations of 20, 50, 100, and $200\,\upmu$g/mL.

The TEM images revealed SPION-DX with approximately spherical shape and mean diameters around $5.9 \pm 1.7$ nm (Fig. 1a and Table 1). This roughly corresponds to the size of the iron oxide core, as the organic coatings are not clearly identifiable in the TEM images. GNPs show a rounded shape with diameters of $5 \pm 2$ nm. The mean hydrodynamic diameters of the NPs in ultrapure water dispersions obtained from light scattering measurements are displayed in Table 1 and are larger than the physical sizes measured by TEM, as expected. The hydrodynamic size is an indication of how the particle behaves in a fluid, considering the hydrated radius around the particle in addition to possible aggregation effects.

The zeta potential ($\zeta$) of the different NPs dispersed in ultrapure water at pH = 7.4 is also given in Table 1 and are typical values usually found for such NP configurations. GNPs  exhibit a negative zeta potential due the hydroxyl terminations from the reduction reaction with borohydride, while SPION-DX show a positive zeta potential, due the amino termination^29–31^. Thermal gravimetric analysis (TGA) was employed to access the mass fraction of the organic coating of the SPION-DX preparation (details in Oliveira et al.^29^). About 78% of the mass of the SPION-DX is composed by the dextran coating and 22% by the Fe$_3$O$_4$ core. Considering that the Fe content in magnetite is about 73% of its mass, we estimate that the dextran mass per nanoparticle is, on average, about five times the mass of Fe. Assuming a density close to 1.8g/cm$^3$ for dextran^32^, a coating layer thickness of about 3.7 nm is estimated.

**Table 1:** Average physical (D) and hydrodynamic (D$_h$) diameters and zeta potential ($\zeta$) of the NPs. TEM data were obtained from direct counting of individual particles. Hydrodynamic diameters and zeta potential were obtained from DLS measurements of NP dispersions in ultrapure water at physiological pH (7.4).

Nanoparticle	D (nm)	D$_h$ (nm)	$\zeta$ (mV)
GNP	$5.2 \pm 2.0$	$7.3 \pm 1.4$	$-15.1 \pm 1.5$
SPION-DX	$5.9 \pm 1.7$	$21.1 \pm 0.9$	$+10.1 \pm 1.1$

### Nanoparticle internalization and citotoxicity

To investigate nanoparticle uptake by cells and their intracellular location, U87 and M059J GBM cells were incubated with different concentrations of well dispersed GNPs and SPION-DX for 24 h. TEM images (Fig. 1b) reveal that NPs were located within the cytoplasm in a heterogeneous distribution. NPs are usually present as aggregates on the order of hundred nanometers which may or may not be inside vesicles. A few isolated NPs were also observed dispersed within the intercellular space and no particles were found in the nucleus. Cell morphology was not altered by the exposure to NPs. Previous works also reported the tendency of metal-based NPs to form aggregates when they are internalized by cells, usually in endo/lysosomal entrapment, restricting nuclear entry^33–43^.

ICP-MS data on cellular metal concentration indicate, however, differences in uptake between cell lines and NP treatments, as shown in Fig. 1c. The uptake of GNPs was proportional to the treatment concentration. Au uptake increases with increasing concentration for both U87 and M059J cell lines. In the case of SPION-DX, the uptake of Fe was different for each cell line considered. For M059J, no significant difference in Fe content within the cells was seen, independent of the initial treatment concentration. On the other hand, significant differences in Fe uptake were observed for U87 cells exposed to $20\,\upmu$g/mL when compared to treatments at higher concentrations. Still, Fe content was similar for cells treated with 50 and $100\,\upmu$g/mL, suggesting a saturation in the internalization capacity of the cells at high NP concentrations. Saturation in Fe uptake has also been found in vivo in zebrafish larvae exposed to similar SPION-DX nanoparticles^44^, but not when U87 cells were treated with iron oxide nanoparticles coated with PEG^45^. Thus, saturation of uptake is not related to the iron oxide itself, but to the coating properties which directly influence the interaction between the cell and the particle^46,47^.

The total mass of Fe taken up by U87 cells was larger than Au for all levels of concentration tested, indicating a higher overall uptake of SPION-DX compared to GNPs (Fig 1c). This trend was also observed for M059J, especially at low exposure concentrations. When comparing uptake between cell lines, U87 showed a considerably higher uptake of SPION-DX than M059J cells ($$p^* = 0.0120$$ for $$20\,\upmu$$g/mL, and $$p^{**} = 0.0011$$ for 50 and $$100\,\upmu$$g/mL). In the case of Au, the uptake was similar for both cell lines, except at $$100\,\upmu$$g/mL for which internalization was higher for U87 cells ($$p^{***} = 0.0002$$).

Cytotoxicity of NPs in the absence of radiation was first evaluated through the MTT assay. This is important to establish the background level of toxicity associated with exposure to the NPs alone. As it can be seen in Fig. 1d, cell viability remained above 95% for all treatment concentrations tested for both NPs. No statistical difference was observed between treated and control groups. This is consistent with previous reports of citotoxicity of GBM cells exposed to GNPs and SPIONs ^45,48,49^. Ahmad et al.^48^ observed no reduction in cell viability even when U87 cells were exposed to a much higher NP concentration ($500\,\upmu$g/mL of Aurovist, AguiX, and SPIONs).

### Radiosensitization effect

**Figure 2:**
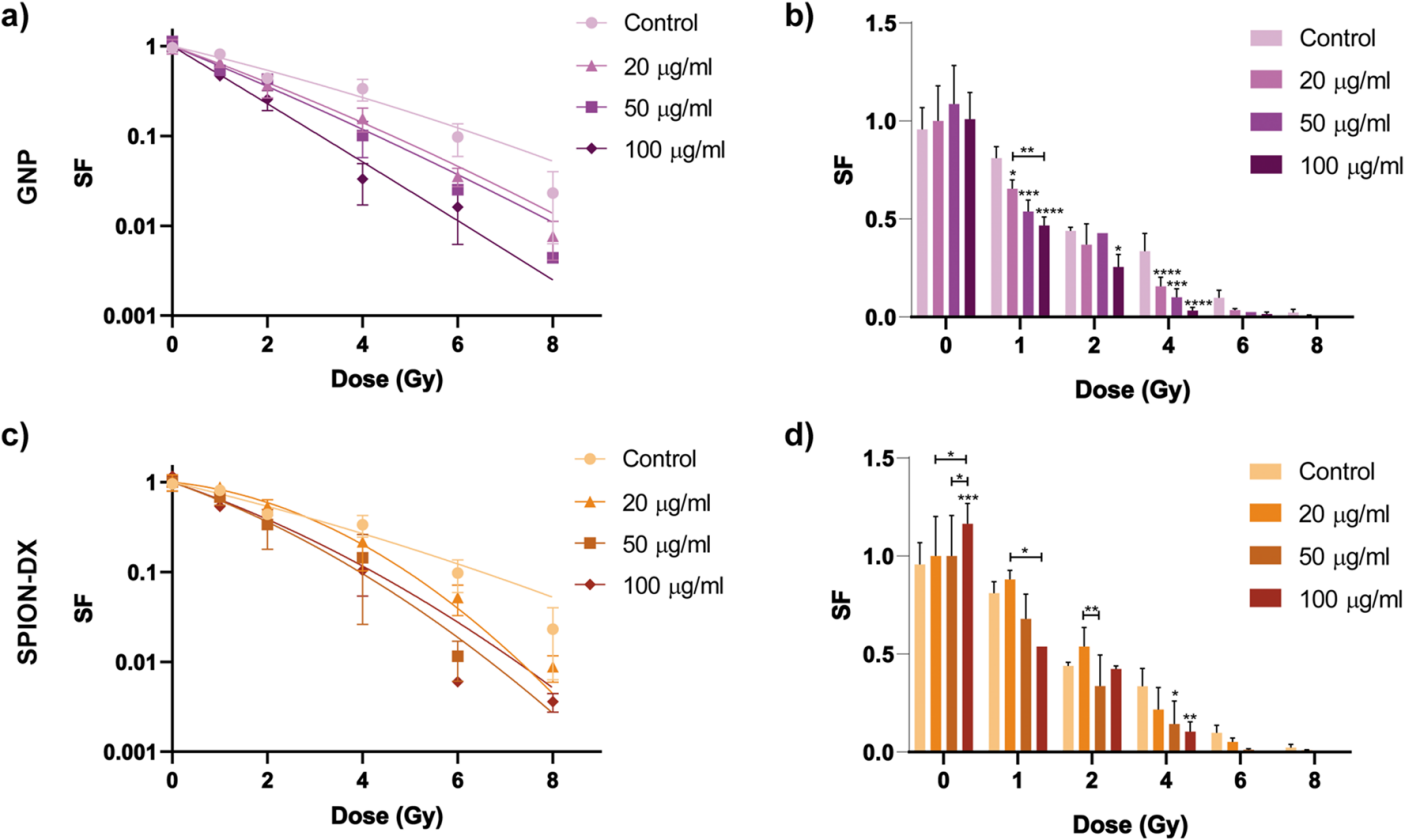
Cell survival curves for U87 GBM cells treated with GNPs(**a,b**), and SPION-DX(**c,d**) at concentrations of 20, 50 and $100\,\upmu$g/mL. Data for untreated cells (control) are also shown.. Samples were irradiated by photons from a 6 MV Linac accelerator in triplicate. The same data was plotted as bar graphs for a better visualization of single dose effects. *p*-values are presented as: *$p < 0.05$; **$p < 0.01$; ***$p < 0.001$; ****$p<0.0001$.

**Figure 3:**
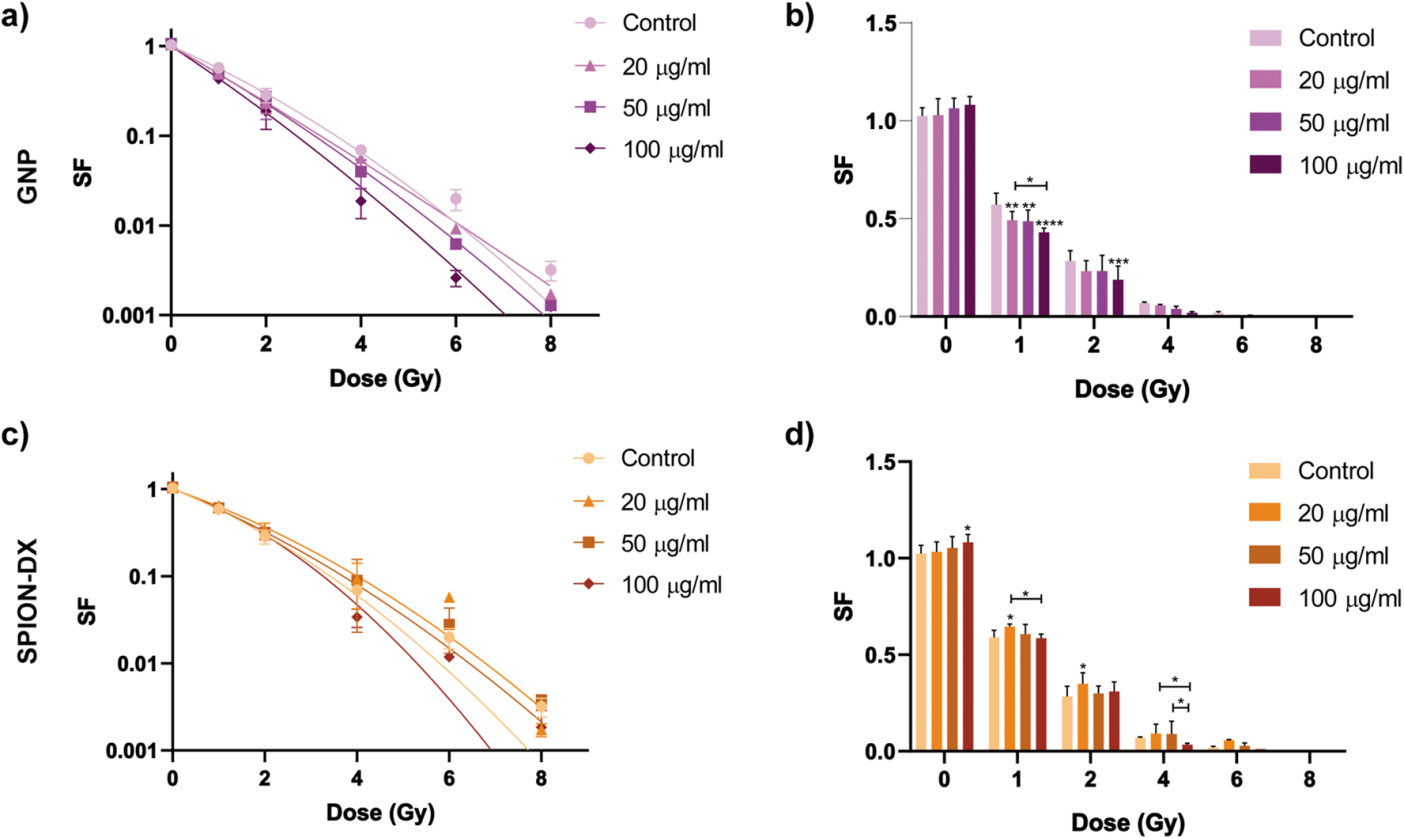
Cell survival curves for M059J GBM cells treated with GNPs (**a,b**), and SPION-DX (**c,d**) at concentrations of 20, 50 and $100\,\upmu$g/mL. Data for untreated cells (control) are also shown. Samples were irradiated by photons from a 6 MV Linac accelerator in triplicate. The same data was plotted as bar graphs for a better visualization of single dose effect. *p*-values are presented as: *$p < 0.05$; **$p < 0.01$; ***$p < 0.001$; ****$p < 0.0001$.

Cell survival was quantified by standard clonogenic assays, and the resultant survival fraction (SF) for U87 and M059J cells are plotted as a function of dose in Figs. 2 and 3, for all different treatment conditions. Survival was also plotted as bar graphs to allow a clearer evaluation of statistical analysis for each irradiation dose. The overall NP effect on cell death, expressed in terms of the sensitization enhancement ratio at a 10 % survival (SER$_{10\%}$) and the $\alpha$ and $\beta$ values were extracted from the cell survival curves. Fold change in the $\alpha /\beta$ ratios between treated and untreated groups were also obtained and used as an indicator of the relative importance of lethal and potentially lethal damage to the cells^9,50,51^. All such parameters extracted from the survival curves are displayed in Table 2.

**Table 2:** Sensitization enhancement ratios calculated at 10% survival (SER$_{10\%}$) for cells irradiated by 6 MV photons after being pre-incubated during 24 h with GNPs and SPION-DX at 20, 50 and $100\,\upmu$g. Fitting parameters ($\alpha$ and $\beta$) were calculated based on the LQ model. Fold change in $\alpha /\beta$ ratio is defined as the ratio of $\alpha /\beta$ derived from the samples treated with NP to the $\alpha /\beta$ extracted from control (untreated) samples.

Cell line	Nanoparticle	Concentration	SER$_{10\%}$	$\alpha$	$\beta$	Fold change in $\alpha / \beta$ ratio
U87	Control	–	1.0	0.2900	0.009821	–
	GNP	$20\,\upmu$g/ml	1.26	0.4450	0.009821	1.33
		$50\,\upmu$g/ml	1.46	0.5042	0.007871	2.17
		$100\,\upmu$g/ml	2.04	0.7341	0.001805	13.78
	SPION-DX	$20\,\upmu$g/ml	1.15	0.1218	0.06943	0.06
		$50\,\upmu$g/ml	1.61	0.4427	0.03845	0.39
		$100\,\upmu$g/ml	1.49	0.4155	0.03039	0.46
M059J	Control	–	1.0	0.4446	0.03675	–
	GNP	$20\,\upmu$g/ml	1.08	0.7057	0.03391	1.72
		$50\,\upmu$g/ml	1.13	0.6807	0.01900	2.96
		$100\,\upmu$g/ml	1.30	0.6507	0.02444	2.20
	SPION-DX	$20\,\upmu$g/ml	0.87	0.4285	0.03613	0.98
		$50\,\upmu$g/ml	0.95	0.4964	0.04142	1.0
		$100\,\upmu$g/ml	1.06	0.4431	0.06441	0.57

For U87 GBM cells treated with GNPs (Fig. 2a and b), the presence of NPs leads to an enhancement on radiation-induced cell killing compared to the untreated groups at all levels of concentration tested. It is noteworthy that the radiosensitization induced by the NPs is shown to be a synergistic effect, since nanoparticles alone have no effect on cell survival. A particularly strong and statistically significant reduction in survival was observed at 1 Gy ($$p^* = 0.0105$$ for $$20\,\upmu$$g/mL, $$p^{***} = 0.0001$$ for $$50\,\upmu$$g/mL, and $$p^{****}< 0.0001$$ for $$100\,\upmu$$g/mL) and 4 Gy ($$p^{****}< 0.0001$$ for 20 and $$100\,\upmu$$g/mL, and $$p^{***} = 0.0001$$ for $$50\,\upmu$$g/mL). This effect is reflected in the high values of SER$$_{10\%}$$, which reached 2.04 at the largest concentration tested. The SER values found here are considerably higher than those reported in other studies involving glioblastomas. For example, Ahmad et al.^48^ reported a SER$$_{10\%}$$ of 1.06, even though they used a much higher treatment concentration of $$500\,\upmu$$g/mL. Values of SER$$_{10\%}$$ comparable to ours (1.91 for T98G cells and 1.3 for U251 cells) were obtained only when low energy X-rays of about 150 keV were used, for which the absorption cross section and thus local dose enhancement is much higher than for MV photons^52,53^. To the best of our knowledge, higher values of SER$$_{10\%} = 2.88$$ were only found for HeLa cells, treated with GNPs and exposed to kVp X-rays^37^. Recent reviews of in vitro studies of cell death enhancement by GNPs and their respective SER$$_{10\%}$$ values can be found elsewhere^9,54^.

In the case of U87 treated with SPION-DX (Fig. 2c,d), the presence of NPs also led to an increase in radiation-induced cell death in comparison to the control, although the effect in cell survival is more complex than what is seen for GNPs . First, the treatment with SPION-DX alone has a positive impact in U87 cell survival. The ability of unirradiated cells to form colonies is improved in the group exposed to the highest NP concentration, resulting in a SF greater than 1.0, as seen in Fig. 2. Significant differences in SF at 0 Gy between the $$100\,\upmu$$g/mL group and the others were observed ($$p^{****} = 0.0005$$ for control, $$p^* = 0.0234$$ for $$20\,\upmu$$g/mL, and $$p^* = 0.0494$$ for $$50\,\upmu$$g/mL). The cause of this effect is at present unclear, but we speculate that it could be connected to the dextran coating, as has been already reported in a few studies with other cell lines^55–57^. For example, dextran supplementation increased the survival of endothelial cells^55,56^. In another study, Easo and Mohanan^57^ showed an increase in levels of lymphocyte proliferation (3–6%) after incubation with dextran coated iron oxide nanoparticles. This favorable consequence of NP exposure may initially counterbalance their radiosensitization effect, but SER$$_{10\%}$$ values for SPION-DX are still relatively large. The highest SER$$_{10\%}$$ observed is 1.61 at the concentration of $$50\,\upmu$$g/mL (Table 2). This is superior to the value of 1.26 reported in U87 cells irradiated with 6 MV x-rays and treated with SPION-DX using a much larger concentration of $$500\,\upmu$$g/mL^48^.

**Figure 4:**
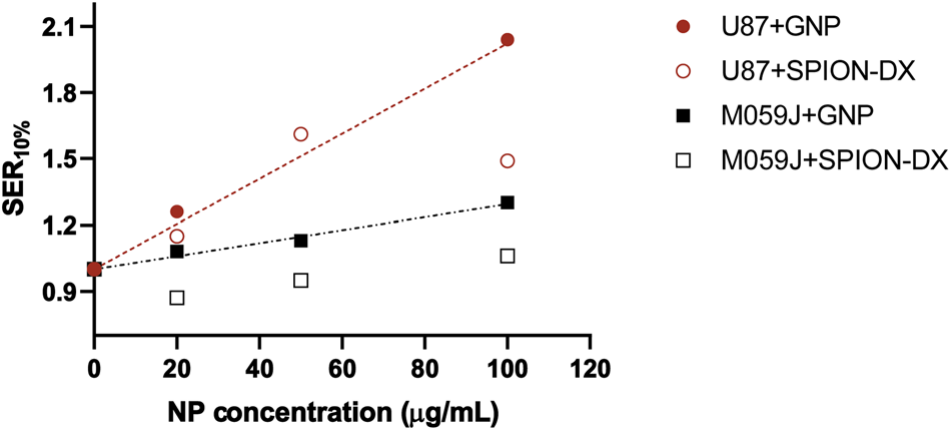
SER$_{10\%}$ as a function of treatment concentration for U87 and M059J cells exposed to GNPs (circles) and SPION-DX (squares). The lines are linear fittings to the GNP experimental points.

In addition, contrary to what is seen for GNPs, the enhancement effect of SPION-DX tend to saturate at high treatment concentrations (Fig. 4). Actually, the SER$_{10\%}$ of 1.49 obtained in the $100\,\upmu$g/mL-SPION-DX treatment, is slightly lower than in the $50\,\upmu$g/mL group. This behavior is consistent with the Fe uptake data (Fig. 1c), which also saturates at high treatment concentrations. Hence, for both gold and iron oxide nanoparticles, a coherent correlation is found between the sensitization enhancement and the actual metal content seen in the cells.

Such result highlights the importance of measuring the actual NP uptake in order to extract meaningful enhancement factors in radiosensitization experiments. Providing only exposure levels is clearly insufficient. However, quantitative analysis of the actual elemental incorporation in cells is often neglected. The widely varying SER values encountered in the literature^9,54^, derived in some cases from seemingly similar experimental conditions, may in part reflect negligence in this aspect.

Next, we explore information that can be derived from the shape of the survival curves. The survival curves of U87 cells exposed to SPION-DX present a shoulder at low doses, while those of GNPs are more straight in the log-linear plot. Such differences are better evaluated from the values of $\alpha$ and $\beta$ parameters and the corresponding $\alpha /\beta$ ratios. Curtis et al.^58^ provided a radiobiological significance for such parameters in his LPL (lethal, potentially-lethal) repair model. According to Curtis‘s model, the non-repairable lesions produce single-hit lethal effects associated with the linear component of survival fraction [exp(-$\alpha$D)]. The repairable lesions depend on the competition between repair and binary misrepair processes, leading to the quadratic component in cell survival [exp(-$\beta$D$^2$)]. Therefore, $\alpha /\beta$ ratio is frequently used to correlate the relative importance of lethal and repairable lesions in the observed radiation effect.

For U87 cells, incubation with SPION-DX led to a significant decrease in the $\alpha /\beta$ ratio relative to the control value. Fold change varies from 0.06 to 0.46 depending on the treatment concentration (Table 2). Thus, based on the the LPL scenario the sensitization induced by SPION-DX occurs mainly via the enhancement of repairable or indirect damage to the cells. The local dose enhancement expected for iron oxide NP for MV photons is considered to be negligible due to their low effective Z^12,59–61^, but iron oxide may still catalyse ROS production in the cells^27^, resulting in chemically driven sensitization effects. Similar inferences have been suggested by other works^27,62,63^.

In contrast, the presence of GNPs induced an increase in $\alpha /\beta$, suggesting the predominance of lethal damage in the enhancement effect. Moreover, higher concentrations of Au led to increasingly steeper SF curves. Fold change in $\alpha /\beta$ ratio increased from 1.33 at $20\,\upmu$g/mL to 13.78 at $100\,\upmu$g/mL (Table 2). There are two probable causes for the observed steepening of SF curves in the presence of GNPs. One is that NPs augment substantially ROS production, inducing clusters of complex and lethal DNA damage^64–73^ and this effect would be more pronounced the higher the NP concentration. In addition, NPs may inhibit DNA repair processes, leading to an increase in residual DSBs^74,75^. We note that a fold change in $\alpha /\beta$ of only 3.4 has been reported for U87 cells treated with $500\,\upmu$g/mL GNPs^48^, in spite of the much higher NP concentration used in that work.

Figure 3 shows the SF curves and bar graphs for M059J GBM cells exposed to GNPs and SPION-DX and irradiated with 6 MV photons. Treatment with GNPs results in sensitization of the cells, although the effect is less pronounced than what is observed for the U87 cell line. For M059J the highest SER$_{10\%}$ is equal to 1.30, roughly 40% lower than the maximum SER for U87 (in spite of the higher Au mass per cell found in M059J cells). As seen in Table 2, the radiosensitization effect is dependent on GNPs concentration. Indeed, for both cell lines the SER$_{10\%}$ values increase linearly with the treatment concentration (Fig. 4) and correlates with a statistically significant increase in the amount of Au detected in the cells with increasing treatment concentration (Fig. 1). In addition, GNPs induced an increase in $\alpha /\beta$ ratios, where fold change for 20, 50, and $100\,\upmu$g/mL were 1.72, 2.96, and 2.20, respectively, again indicating the predominance of lethal damage in the enhancement effect elicited by GNPs.

On the other hand, treatment with SPION-DX at all concentration tested did not result in any significant biological effect in the irradiated M059J cells. As seen for U87, the treatment with a high concentration of SPION-DX improved survival of unirradiated M059J cells in respect to the control group ($p^* = 0.033$). This effect appears also in cells incubated with $20\,\upmu$g/mL and irradiated with 1 and 2 Gy (Fig. 3d). Consequently, SER$_{10\%}$ values are actually smaller than one for the treatment concentration of $20\,\upmu$g/mL, being slightly above the control level only at the highest concentration used (Fig. 4). Changes in $\alpha /\beta$ ratio are also negligible, except for the 0.57-fold change seen in the group exposed to $100\,\upmu$g/mL SPION-DX(Table 2). We note that Fe uptake by M059J cells was similar in all treatments, showing no significant differences among groups exposed to nanoparticle concentrations of 20, 50, and $100\,\upmu$g/mL (Fig. 1c). This may in part explain the weak sensitization effects observed, even at high levels of NP exposure.

Overall, the sensitizing effect induced by both NPs is more pronounced in the radioresistant cell line (U87) than in the radiosensitive M059J, as can be seen in Fig. 4. This is clearly evident in the case of GNPs treatments, where the SER$_{10\%}$ increase per mass of GNPs added is three times greater for U87 than M059J cells. Those numbers are extracted from the slope of linear fittings to the SER$_{10\%}$ versus NP concentration curve (solid and dashed lines in Fig. 4), which is about 0.01 ($\upmu$g/mL)$^{-1}$ for U87 and 0.003 ($\upmu$g/mL)$^{-1}$ for M059J.

## Conclusion

In this work, an intercomparison of radiosensitization effects produced by gold and dextran-coated superparamagnetic iron oxide nanoparticles in M059J and U87 human glioblastoma cell lines was performed using 6 MV photons. Three different variables were mapped: the NP material, the treatment concentration, and the cell radiosensitivity. For U87 cells, GNPs treatments resulted in high sensitization enhancement ratios, reaching a 2-fold increase, superior to values reported previously. More modest effects were induced by SPION-DX, but still significant reduction in cell survival was achieved, with a maximum SER$_{10\%} = 1.61$ in the group exposed to $50\,\upmu$g/mL of nanoparticles. For the radiosensitive M059J cells, sensitization assisted by both types of nanoparticles was much less efficient.

Qualitative differences were also found in the effect of NP treatments in the shape of the survival curves, which were quantified through the fold change in the $\alpha /\beta$ ratio. Fold change is reduced by the presence of SPION-DX and strongly increased in cells treated with GNPs, suggesting that sensitization by GNPs occur mainly via the promotion of lethal complex damages, but for SPION-DX repairable damage dominates. Work is in progress to investigate in more detail such differences in the sensitization mechanism, including the impact of NPs in cellular signalling, quantification of ROS and DNA damage/repair, which will be reported elsewhere.

In addition, we demonstrate that, for both nanoparticles and cell lines, the sensitization enhancement ratio increases proportionally to the actual degree of metal internalization in the cell, but not necessarily with the concentration of NPs during incubation. This is an important aspect to consider, as quantitative analysis of the elemental incorporation in cells is often not provided. For Au treatments, where exposure levels and elemental uptake were found to be proportional, SER$_{10\%}$ increased linearly with concentration in both cell lines. However, the SER$_{10\%}$ increase per mass of GNPs added is three times greater for U87 than M059J cells. For SPION-DX saturation of both sensitization enhancement and metal uptake occurred at high levels of exposure.

Overall, the combination of NPs with radiotherapy was more effective in eliminating radioresistant than radiosensitive GBM cells, an interesting finding, as radiosensitization of resistant cells is an important target to improve treatment outcome in patients. To ensure that this is not a peculiarity of the cell line it would be worth investigating in future work whether cell killing in other types of radiosensitive cancer cells is equally poorly affected by the action of nanoparticles, as seen here.

## Methods

### Nanoparticle synthesis and characterization

High-purity water (resistivity of 18.6 M$\Omega$-cm) was employed in all synthesis. The reagents used were all of analytical grade, acquired from the following brands: a) Merck (ferrous and ferric chloride, ammonium hydroxide (25%), and sodium hydroxide); b) SigmaAldrich (chlorouric acid, sodium borohydride, and epichlorohydrin); c) Pharmacosmos (Pharmacosmos (polysaccharide dextran T10 with average molecular weight of 10.000 Da).

Gold nanoparticles (GNPs) were synthesized via the reduction of chlorouric acid (HAuCl$_4$.3H$_2$O) by sodium borohydride (NaBH$_4$), following a method adapted from Deraedt et al.^76^. Aminated dextran-coated superparamagnetic iron oxide nanoparticles (SPION-DX) were prepared from a mixture of Fe (II) and Fe (III) salts, according to Oliveira et al.^29^ and following the method of coprecipitation with nucleation of nanoparticles directly in the presence of dextran^77^. An additional crosslinking step of the dextran shell was performed by adding 5 M NaOH and 14 mL of epichlorohydrin into the NPs solution, under magnetic stirring for 10 h. Finally, for amination of the coating, 60 mL of NH$_4$OH (28%) was added to the solution and maintained under magnetic stirring for 24 h. The ammonia excess was extracted by dialysis, using Spectra/Por$\circledR$ membranes and changing the deionized water every 30 min. At the end, both nanoparticle dispersions (GNPs and SPION-DX) were washed and centrifuged in amicon tubes (50k MWCO) for 15 min several times to eliminate undesired residues derived from the synthesis. The final stock solutions were stored at 4 $^{\circ }$C. SPION-DX were kept in a sodium citrate buffer solution.

The analysis of the size distribution and morphology of NPs were performed by transmission electron microscopy (TEM) and dynamic light scattering (DLS). For TEM observations a Tecnai G2 T20 - FEI was used. A drop of the NP suspension was dripped on carbon film TEM grids, and left at room temperature until dry. The grids were kept in vacuum for at least 24 h before analysis. The average diameter of the NPs was measured using the ImageJ software, counting at least 100 individual particles for each formulation. Additional TEM images of the SPION-DX at higher magnifications are available in the Supplementary Material Figure S1. The measurements of the hydrodynamic diameter and the zeta potential ($\zeta$) of the NPs in aqueous solution at physiological pH (7.4) were performed in a Zetasizer, model ZEN3600-Malvern, at room temperature. Ultra-pure deionized water was used to dilute the stock solution to a concentration of 10 mM.

### Cell culture and exposure to nanoparticles

Human U87 and M059J GBM (from ATCC) cells were cultured in Dulbecco’s Modified Eagle medium (DMEM) (Gibco, Life Technologies) supplemented with 10% fetal bovine serum (FBS) and 1% penicillin/streptomycin. Cells were maintained in a cell incubator at $37\,^\circ \hbox {C}$, 5% CO$_2$, and 95% humidity. The cells were exposed to GNPs and SPION-DX dispersed in the culture medium at concentrations of $20\,\upmu$g/mL, $50\,\upmu$g/mL, and $100\,\upmu$g/mL during an incubation time of 24 h.

### Intracellular distribution of nanoparticles

TEM was performed to identify the intracellular distribution of NPs. After exposure to the NPs, cells were fixed for 2 h with a solution containing 2.5% glutaraldehyde, 2% parafolmadeide, and phosphate buffer. After fixing, the samples were washed three times with 0.1 M phosphate for 30 min. Post-fixation was done with Osmium tetroxide and 0.2 M phosphate buffer for 45 min and washed again. Then, the samples were dehydrated with acetone and soaked in resin for 24 h. The cells already embedded in pure resin were left in the oven at a constant temperature of 60 $^{\circ }$C for 72 h. Finally, the samples were cut by ultramicrotomy into 100 nm slices and deposited in TEM grids for imaging in a FEI Tecnai G2 T20 microscope.

### Quantification of elemental cellular uptake

Quantification of elemental cellular uptake was performed by inductively coupled plasma mass spectrometry (ICP-MS, Agilent-2012). Cells were seeded in 12-well plates and treated with NPs as described before. Following the incubation period of 24 h, the NPs were removed, and the wells were gently washed twice with PBS. Cells were trypsinized and counted using trypan blue to determine the total number of cells per sample. The cell suspension was centrifuged for 5 min at 1000 RPM to produce pellets which were then dissolved with aqua regia (three parts hydrochloric acid to one-part nitric acid). The final solution was diluted with ultrapure water. Reference measurements were also carried out on a known concentration of each metal, to obtain a calibration curve relating the ICP counts to the metal concentration. Each sample was then processed and counts were related to the reference curve to determine the elemental concentration per sample. Results were then reported as the mass of metal (Au or Fe) per cell (pg per cell). Based on ICP mass quantification and considering the nanoparticles as spherical, and with the same density as in the bulk, one may also estimate the cellular uptake in terms of nanoparticle concentration. Uptake values in units of NP/cell can be obtained multiplying the cellular uptake in pg/cell by the conversion factor of $6.85 \times 10^{5}$ for GNPs and $2.46 \times 10^{6}$ for SPION-DX. Similar factors can be used to estimate the treatment concentration in NP/mL. For example, $20\,\upmu$g/mL correspond to $4.93 \times 10^{13}$ NP/mL for SPION-DX and $1.37 \times 10^{13}$ NP/mL for GNPs.

### Cell viability assay

Cell viability was determined by MTT (3-4,5-dimethyl-thiazol-2-yl-2,5-diphenyltetrazolium bromide) assay. Cells were seeded in 96-well plates at $3\times 10^3$ cells/well. The cells were then incubated with the NPs at concentrations ranging from $20\,\upmu$g/mL to $200\,\upmu$g/mL. After 24 h, NP solutions were removed and $100\,\upmu$L of the MTT solution in PBS (0.5 mg / mL) was added and incubated for 2 h. The MTT solution was removed and replaced with DMSO, $100\,\upmu$l/well. The color intensity of the formazan solution, which reflects cell viability, was measured at 570 nm using a Spectra Max M2e (Soft Max$\circledR$ Pro 5, Molecular Devices).

### Cell irradiation

Photon irradiations were performed in the Radiotherapy Center of São Lucas Hospital at PUCRS in a 6 MV clinical accelerator (Clinac IX and Clinac Trilogy by Varian). To perform the irradiations, we have developed an acrylic sample holder of 30 cm $\times$ 30 cm with a slot to fit the culture plates in the central region. The fitting was tight enough to minimize the air between the plate and the walls of the holder, but still allowing easy removal of the plates. In addition, 5 cm of solid water was positioned on top of the holder for radiation build up. Another layer of 3 cm solid-water bolus was placed under the sample holder to simulate backscattered radiation. Prior to irradiation, a tomographic image of the cell plate inserted in the sample holder was performed using the three-dimensional planning software Eclipse, in order to determine accurately the dose at the region of interest. For further details of the irradiation set-up, see Supplementary Material Figure S2.

The irradiations were carried out in a $20\times 20$ cm$^2$ field with a SSD of 93 cm, and dose rate of 1 Gy/min. The size of the field was chosen so that the isodose curves were as uniform as possible. Cells to be irradiated were seeded in 12-well plates at $10^5$ cells / well and incubated with the NPs for 24 h, as described previously. After the incubation time, the cells were irradiated with doses of 1, 2, 4, 6 and 8 Gy. For each irradiation experiment, an untreated group was transported to the irradiation site, as a control group. All irradiations were performed at a confluence of 70- 80%.

### Clonogenic assay

Clonogenic assays were performed to evaluate the survival fraction (SF) of the irradiated cells, following the procedure described in^78^. After irradiation, cells were detached from the plates using trypsin / 0.05% EDTA for 5 min. Cells were counted and seeded in triplicates in 6-cm dishes at the appropriate concentrations, depending on the irradition dose. After 10–14 days, colonies were fixed and stained with a mixture of glutaraldehyde and crystal violet for 30 min. Colonies containing at least 50 cells were counted. All survival fractions were normalized for control plating efficiency (0 Gy). The sensitization enhancement ratio at a 10 % survival (SER$_{10\%}$)^5,50,51^ was derived to quantify the effectiveness of the NPs action:

1 $$\begin{aligned} SER_{10\%} =\frac{Dose\,at\,10\% \,survival\,without\,NPs\,(D_{control})}{Dose\,at\,10\% \,survival\,with\,NPs\,(D_{NPs})} \end{aligned}$$

SF curves as a function of dose (D) were also obtained and fitted to the linear-quadratic (LQ) model

2 $$\begin{aligned} SF(D) = exp (-\alpha D - \beta D^2), \end{aligned}$$

to extract $\alpha$ and $\beta$ parameters for each treatment condition.

### Statistical analysis

All the experiments were repeated in triplicate on separate days. Curve fittings were performed with the software GraphPad Prism. To evaluate the statistical differences between the experimental and corresponding control samples, the data were analyzed using analysis of variance (ANOVA) with post-hoc Bonferroni correction. $p < 0.05$ was considered significant.

## Supplementary Information

Supplementary Information.
