# Supplementary material for: Intercomparison of radiosensitization induced by gold and iron oxide nanoparticles in human glioblastoma cells irradiated by 6 MV photons
Source: Sci Rep. 2022 Jun 10;12:9602. doi: 10.1038/s41598-022-13368-x (PMC9187689; doi:10.1038/s41598-022-13368-x)
Supplement: Supplementary file 1 — Supplementary Information. [file 41598_2022_13368_MOESM1_ESM.pdf]

Supplementary Material for "Intercomparison of radiosensitization induced by gold and iron oxide nanoparticles at different concentrations in human glioblastoma cells irradiated by 6 MV photons "

Danieli B. Guerra[1*], Elisa M. N. Oliveira[1], Amanda R. Sonntag[1], Patricia Sbaraine[2], Andre P. Fay[3], Fernanda B. Morrone[4] and Ricardo M. Papaléo[1]

[*]Correspondence: danieli.guerra@acad.pucrs.br

[1] Interdisciplinary Center for Nanoscience and Micro-Nanotechnology, School of Technology, Pontifical Catholic University of Rio Grande do Sul, PUCRS, Porto Alegre, Brazil.

[2]Division of Radiotherapy, São Lucas Hospital of PUCRS, Porto Alegre, Brazil.

[3]School of Medicine, Pontifical Catholic University of Rio Grande do Sul, PUCRS, Porto Alegre, Brazil.

[4]School of Health and Life Sciences, Pontifical Catholic University of Rio Grande do Sul, PUCRS, Porto Alegre, Brazil.

Transmission Electron Microscopy (TEM) images of SPION-DX.

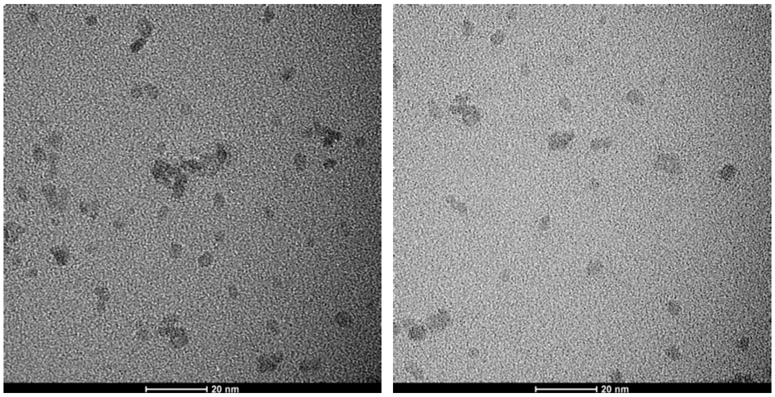

Figure S1. TEM images of SPION-DX at high magnifications (× 6300k).

Details of the irradiation set up.

X-ray irradiations were performed in the Radiotherapy Center of Hospital São Lucas at PUCRS in a 6 MV linear clinical accelerator (Clinac IX and Clinac Trilogy by Varian). To perform the irradiations, we developed a rectangular acrylic phantom (30cm X 30cm) with a slot for the culture plates in the central region (Figure S1 a and b). The phantom was made so that the plate can be easily removed, but still minimizing the volume of air between the plate and the walls of the phantom. In addition, a 3 cm solid water bolus was placed under the phantom/plate system to simulate backscattered radiation. Another layer of 5 cm of solid water is positioned between the plate and the exit of the X-ray beam for build-up.

The irradiation planning was done based on tomographic images of the phantom, using the three-dimensional planning software Eclipse, as represented in Figure S1 d. The irradiations were carried out with a 20 X 20 cm field and a source to surface distance SSD = 93 cm . The size of the field was chosen so that the isodose curves were as uniform as possible in the region of interest.

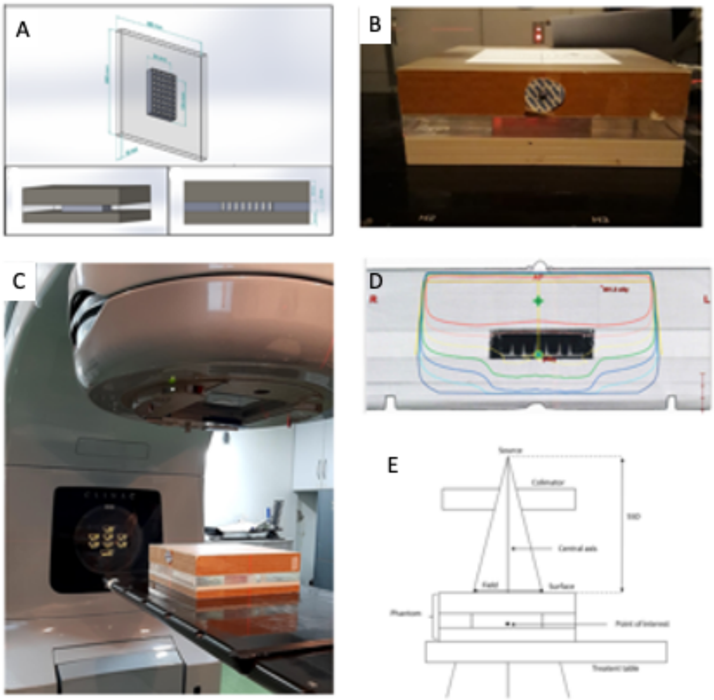

Figure S2. Irradiation set up. A) Scheme of the acrylic phantom constructed for the irradiation of the cell culture plates. B) The phantom-plate system is positioned between two boluses of solid water, one 5 cm -thick placed on the top and another 3 cm-thick positioned below the phantom. C) Irradiation set-up at the radiotherapy center, Hospital São Lucas da PUCRS. The phantom is placed in the 6 MV Linac accelerator. D) Transverse tomographic image of the phantom, where the coloured contour lines represent the isodose curves. The central region (isocenter) was placed at the position of the cell culture plate. E) Representative scheme of the irradiation set up. The “x” in the center defines the region of interest, which coincides with the isocenter. The irradiation field (20 x 20 cm) and source to surface distance (SSD =93 cm) are depicted.
